# A Rare Case of Metastatic Desmoplastic Small Round Cell Tumour: Diagnosis and Management

**DOI:** 10.1155/2015/925453

**Published:** 2015-08-09

**Authors:** Shahzaib Nabi, Abhijit Saste, Rohit Gulati

**Affiliations:** ^1^Department of Internal Medicine, Henry Ford Health System, 2799 W. Grand Boulevard, Detroit, MI 48202, USA; ^2^Department of Hematology-Oncology, Henry Ford Health System, 2799 W. Grand Boulevard, Detroit, MI 48202, USA; ^3^Department of Pathology, Henry Ford Health System, 2799 W. Grand Boulevard, Detroit, MI 48202, USA

## Abstract

A 26-year-old male without any significant past medical history presented to the hospital with shortness of breath, cough, pleuritic chest pain, and weight loss for the past 3 months. On chest CT, he was found to have extensive mediastinal and hilar lymphadenopathy and multiple pulmonary nodules. On physical examination, a right groin mass was noted which had been slowly growing for the past 2 years. Ultrasound of the groin showed complex solid mass with internal vascular channels. CT guided biopsy of the mass showed desmoplastic small round cell tumour. His hospital course was complicated by hypoxic respiratory failure requiring emergent intubation and ICU admission where he completed one cycle of vincristine, cyclophosphamide, and doxorubicin with subsequent improvement, followed by extubation. His condition continued to improve after second cycle of chemotherapy and he was ultimately discharged in a stable condition to continue outpatient chemotherapy after a 2-month inpatient stay.

## 1. Introduction

This case report describes our clinical experience in the diagnosis and management of a rather uncommon malignancy. Given the rarity of this condition, with less than 200 cases reported to date, every case experience such as ours adds to the understanding of how varying clinical scenarios can be successfully managed and what the responses looked like. Additionally, a pool of such cases with information on progression free survivals and response rates may then help future investigators gain a better understanding of the efficacy of such a regimen, safety signals of its components, and management of any complications that clinicians may have encountered. More importantly, we successfully investigated and treated an intubated patient with chemotherapy to then discharge him in an ambulatory condition. He maintains an ECOG performance status of 0.

## 2. Case Presentation

Our patient is a 26-year-old male with no significant past medical history. He presented to the hospital with a 3-month history of shortness of breath, dry cough, and pleuritic chest pain. His symptoms started gradually but started to worsen about a week before presenting to the hospital. The shortness of breath was mainly exertional. Patient was also experiencing a dry cough related to this difficulty of breathing. He also complained of pleuritic chest pain, bilaterally in both lower chest fields, 5/10 in intensity, dull in nature without any radiation. Aggravating factor included bouts of cough but there were no specific relieving factors. The patient had also noticed some unintentional weight loss but was unsure about the amount of lost weight. Review of systems was negative for fever or any exposure to sick contacts. There was no history of night sweats. He did not have any risk factors for tuberculosis. There was no history of any recent travel and the patient did not complain of any leg swelling.

Physical examination showed a thin and lean male in slight respiratory distress. Patient was found to be tachypneic with a respiratory rate of 34 breaths per minute and tachycardic with a heart rate of 118 beats per minute. He was afebrile and normotensive. Auscultation of the lungs revealed diffuse bilateral wheezing. Chest palpation was negative for chest wall tenderness. Auscultation of the heart revealed normal S1 and S2 with no added sounds. Neurologically, the patient was alert and oriented to time, place, and person. He was able to follow commands with no focal neurological deficits. Abdominal examination showed a soft, nontender abdomen with no organomegaly and normal bowel sounds. A large, firm, nontender mass, with poorly defined margins was palpated in the right groin. It was not reducible and there were no signs of infection (no redness, tenderness, or warmth). There was no change in the size or shape of the mass when the patient was asked to perform the valsalva maneuver. According to the patient, the mass had been slowly growing in his groin for the past 2 years. Penile examination was negative for any ulcers or discharge. Scrotal examination did not show any apparent testicular masses.

The patient's family history was negative for any significant problems. The patient did not smoke or drink alcohol and had no risk factors for sexually transmitted diseases. He was not taking any medications at the time of admission.

## 3. Investigations

Complete Blood Count (CBC) and basic metabolic panel were normal.

**Figure 1:**
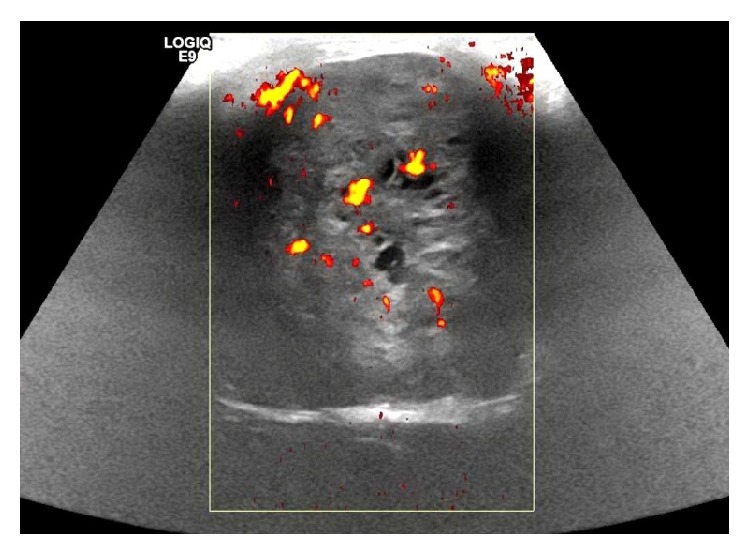
Ultrasound of groin showing complex vascular mass.

**Figure 2:**
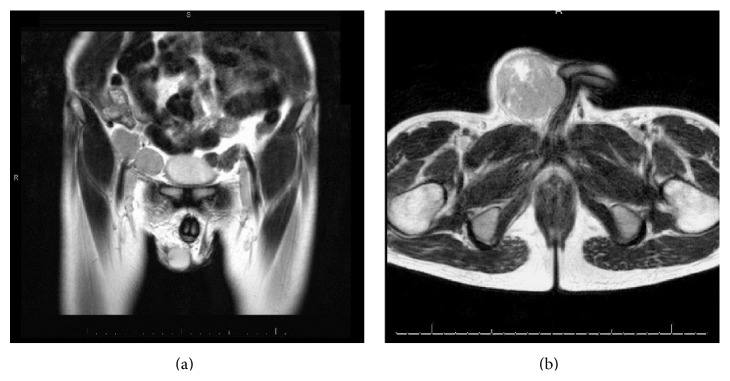
MRI coronal (a) and transverse (b) section showing mass lesion in the right inguinal region with mass effect on the corpora cavernosa of the penis.

Ultrasound of the pelvis and scrotum showed a 7.5 × 5.5 × 5.9 cm complex vascular mass in the rightward mons pubis with unremarkable sonographic appearance of the testicles (Figure 1). MRI of the pelvis showed mass lesions involving the right inguinal canal extending to the distal right iliac chain likely consistent with enlarged lymph nodes. Significant effacement and mass effect was seen upon the right corpora cavernosa of the penis without definite evidence of soft tissue or osseous invasion (Figure 2). Significant narrowing and effacement of the right external iliac vein were seen at the level of mass lesions.

**Figure 3:**
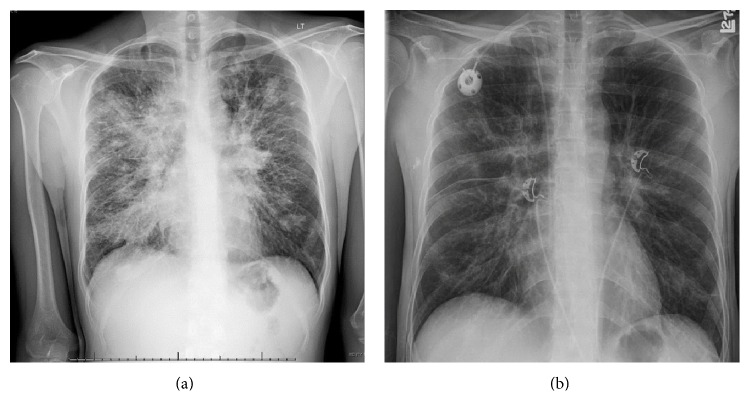
Chest X-ray with evidence of bilateral pulmonary opacities and hilar fullness on presentation (a) and after 4 cycles of VAC/IE chemotherapy (b).

**Figure 4:**
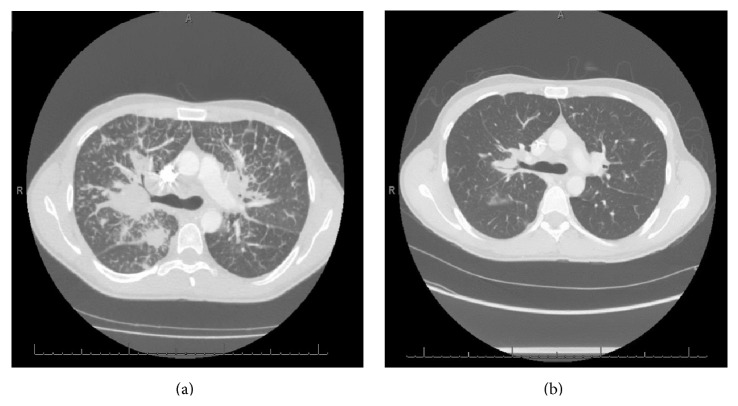
CT chest with large masses bilaterally at level of bifurcation of trachea (a) and after 4 cycles of VAC/IE chemotherapy (b).

Chest X-ray showed extensive parenchymal opacities throughout both lungs with mediastinal fullness (Figure 3). CT scan of the chest showed extensive mediastinal and hilar lymphadenopathy with prominent interstitial changes throughout both lungs and multiple diffuse pulmonary nodules (Figure 4).

**Figure 5:**
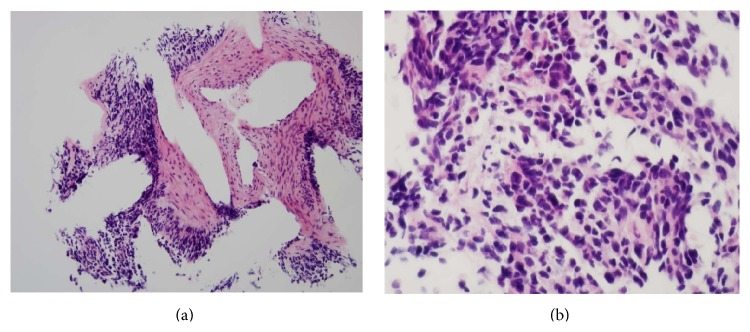
H&E stain (×200 on (a)) showing nests and sheets of small round blue cells infiltrating desmoplastic stroma. Tumour cells (×600 on (b)) with round to oval, hyperchromatic, mitotically active nuclei with scant cytoplasm.

**Figure 6:**
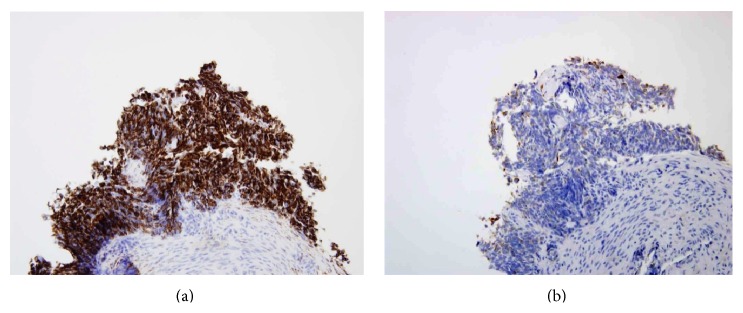
Immunohistochemical staining showing strong positivity for desmin (a) and focal positivity for cytokeratin (b).

**Figure 7:**
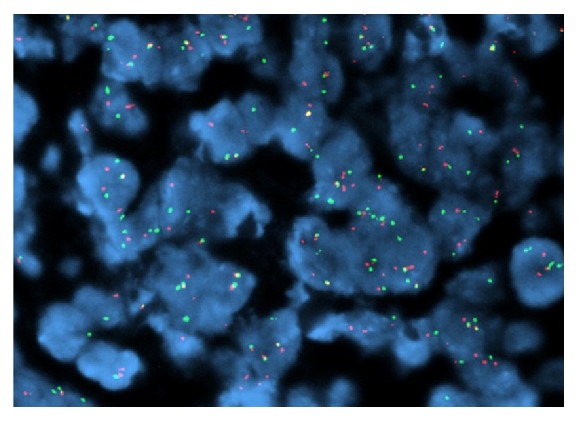
FISH assay showing positivity for EWSR1 gene rearrangement. Sixty five percent of the interphase cells showed separation of EWSR1.

Ultrasound guided biopsy of the inguinal mass was carried out and pathology results were consistent with desmoplastic small round cell tumour (DSRCT) (Figure 5). Immunohistochemical staining showed desmin and cytokeratin (CAM 5.2) positivity (Figure 6). CD56 immunostain and leukocyte common antigen were negative. A fluorescent in situ hybridization (FISH) assay was positive for EWSR1 break apart (Figure 7). RT-PCR was negative for EWS-Fli1 and EWS-ERG rearrangements, indicating absence of t(11;22) and t(21;22), respectively, of Ewing's sarcoma/PNET. Endobronchial ultrasound (EBUS) guided biopsies of the lymph nodes and pulmonary masses were performed. The pathology was consistent with desmoplastic small round cell tumor.

## 4. Treatment

The patient's hospital course was complicated by respiratory distress and hypoxia resulting in admission to the intensive care unit where he was intubated for hypoxic respiratory failure. The etiology was believed to be resorption atelectasis secondary to the bronchial compression from metastatic lesions and a pneumothorax from his malignancy. He had a chest tube placed which successfully treated his pneumothorax. While on the ventilator, he was initiated on and completed cycle 1 of vincristine, cyclophosphamide, and doxorubicin as a part of the Memorial Sloan Kettering Cancer Center P6 chemotherapy protocol of VAC/IE (vincristine, adriamycin, and cyclophosphamide alternating with ifosfamide and etoposide). This resulted in significant regression in his pulmonary tumour burden and subsequent extubation. His course was also complicated by febrile neutropenia which were managed with intravenous antibiotics, transfusions, and growth factor support. He was eventually discharged from the hospital in a stable condition after a 2-month-long inpatient stay.

The patient continues to follow up with the oncology clinic as an outpatient and gets electively admitted for his chemotherapy. He is occupationally functional and maintains a good appetite. He maintains an ECOG performance status of 0. He has completed 3 cycles of vincristine, cyclophosphamide, and doxorubicin followed by two cycles of ifosfamide and etoposide. A restaging CT scan of the chest and abdomen done after completion of radiation and cycle 4 of VAC/IE has shown significant reduction in the size of his groin mass and pulmonary metastases (Figures 3 and 4). He has completed radiation therapy to the right groin mass for a total of 37.5 Gray given as 2.5 Gray daily for a total of 15 fractions. He is currently scheduled to undergo autologous stem cell transplant.

## 5. Discussion

Desmoplastic small round cell tumours (DSRCT) are a rare group of sarcomas found in almost all age groups. Like all other sarcomas, they are of mesenchymal origin. They occur more commonly in adolescents and young adults. They were first described in 1989 by Gerald and Rosai as small round blue cell tumours with predilection for serosal surfaces and occurring predominantly in young Caucasian males with predominantly intra-abdominal locations with focal rhabdoid pattern with an intense desmoplastic reaction [1, 2]. Up till 2013, less than 200 cases had been reported in the world literature [3].

Histologically, the tumour consists of poorly differentiated round cells with cytoplasmic densities and connective tissue stroma. They typically show immunohistochemical positivity for desmin, cytokeratin, vimentin, and CAM5.2 [4]. The peculiar perinuclear dot-like staining pattern for vimentin and desmin is characteristic for DSRCT. These tumours arise from a reciprocal translocation, t(11;22)(p13;q12), which results from fusion of Ewing's sarcoma (EWS) and Wilms' tumour (WT1) genes [5–8]. Histopathology is not sufficient to diagnose DRSCT as certain other tumours such as primitive neuroectodermal tumour can mimic DRSCT under the microscope. Molecular analysis is required for a final diagnosis [9].

The majority of the cases of DRSCT occur in the abdomen; however, many case reports have described this tumour originating from different organs such as testis, extremities, salivary glands, and brain [10–13]. Clinical presentation depends on the primary location of the tumour as well as disease stage. Diagnosis typically requires a tissue biopsy with identification of histopathological features along with immunohistochemistry and molecular testing. Fine-needle aspiration has also been used for the diagnosis of DRSCT [14]. As previously mentioned, hallmark feature in molecular testing is the presence of WT1-EWS fusion; however, case reports with atypical molecular features have also been described [15].

Surgery, radiation therapy, and chemotherapy are modalities that are utilized for the treatment of DRSCT but given the rarity of the condition no large scale prospective trials exist for head to head comparisons. Hyperthermic intraperitoneal chemotherapy using Cisplatin has been utilized as a low morbidity treatment option for DSRCT patients [16–18]. The reported results have been variable with some studies showing survival benefit when used instead of traditional systemic chemotherapy [19–21]. Yttrium microspheres have been used successfully to treat liver metastasis from DSRCT [22]. For limited stage disease, complete surgical resection is the treatment of choice. Based on the tumour size and grade, neoadjuvant/adjuvant radiotherapy and chemotherapy can be utilized.

The Memorial Sloan Kettering Cancer Center P6 protocol is one of the most studied and therefore commonly used chemotherapy regimens for DSRCT. It consists of seven cycles of chemotherapy. Cycles 1, 2, 3, and 6 include high dose cyclophosphamide, doxorubicin, and vincristine (VAC). Cycles 4, 5, and 7 consist of ifosfamide and etoposide (IE). Myeloablative chemotherapy with etoposide and thiotepa, followed by allogeneic stem cell transplantation, has been tried. Studies have shown that intense multimodality approach is associated with better outcomes [23, 24]. Certain investigational drugs, such as temsirolimus, an antiangiogenic serine/threonine protein kinase inhibitor, are being considered for these tumours, but the data on these is very limited [25]. Another agent named pazopanib, a tyrosine kinase inhibitor, is also under investigation for the treatment of these tumours [26].

The 5-year survival rate of DRSCT is only approximately 15% [27]. The prognosis is generally poor as the majority of the patients have metastatic disease at the time of presentation. The median survival ranges from 17 to 25 months [28].
